# Revolutionizing Therapeutic Drug Monitoring with the Use of Interstitial Fluid and Microneedles Technology

**DOI:** 10.3390/pharmaceutics9040043

**Published:** 2017-10-11

**Authors:** Tony K.L. Kiang, Sahan A. Ranamukhaarachchi, Mary H.H. Ensom

**Affiliations:** 1 Faculty of Pharmacy and Pharmaceutical Sciences, University of Alberta, Edmonton, AB T6G 2E1, Canada; tkiang@ualberta.ca; 2 Department of Electrical and Computer Engineering, University of British Columbia, Vancouver, BC V6T 1Z4, Canada; sahan@microdermics.com; 3 Faculty of Pharmaceutical Sciences, University of British Columbia, Vancouver, BC V6T 1Z3, Canada

**Keywords:** clinical pharmacokinetics, therapeutic drug monitoring, interstitial fluid, microneedles

## Abstract

While therapeutic drug monitoring (TDM) that uses blood as the biological matrix is the traditional gold standard, this practice may be impossible, impractical, or unethical for some patient populations (e.g., elderly, pediatric, anemic) and those with fragile veins. In the context of finding an alternative biological matrix for TDM, this manuscript will provide a qualitative review on: (1) the principles of TDM; (2) alternative matrices for TDM; (3) current evidence supporting the use of interstitial fluid (ISF) for TDM in clinical models; (4) the use of microneedle technologies, which is potentially minimally invasive and pain-free, for the collection of ISF; and (5) future directions. The current state of knowledge on the use of ISF for TDM in humans is still limited. A thorough literature review indicates that only a few drug classes have been investigated (i.e., anti-infectives, anticonvulsants, and miscellaneous other agents). Studies have successfully demonstrated techniques for ISF extraction from the skin but have failed to demonstrate commercial feasibility of ISF extraction followed by analysis of its content outside the ISF-collecting microneedle device. In contrast, microneedle-integrated biosensors built to extract ISF and perform the biomolecule analysis on-device, with a key feature of not needing to transfer ISF to a separate instrument, have yielded promising results that need to be validated in pre-clinical and clinical studies. The most promising applications for microneedle-integrated biosensors is continuous monitoring of biomolecules from the skin’s ISF. Conducting TDM using ISF is at the stage where its clinical utility should be investigated. Based on the advancements described in the current review, the immediate future direction for this area of research is to establish the suitability of using ISF for TDM in human models for drugs that have been found suitable in pre-clinical experiments.

## 1. Introduction

Therapeutic drug monitoring (TDM) is broadly defined as the science or practice of optimizing drug therapy by targeting drug concentrations in biological matrices to the defined therapeutic range in order to increase and decrease the probabilities of efficacy and adverse effects, respectively. Fundamentally, the science of pharmacokinetics (PK) is the underpinning for the practice of TDM; that is, how the absorption, distribution, metabolism, and elimination processes of a drug dictate how TDM is implemented. Not all drugs are subjected to TDM and considerable research is continuing to be conducted on all aspects of TDM today.

From the practical stand point, the traditional gold-standard approach to TDM is to collect blood (hereafter, the term “blood” will be used in this manuscript to infer any biological matrices related to blood, plasma, or serum), because it is relatively easier to obtain compared to other matrices (e.g., organ tissues). Due to the fact that blood remains the gold standard for TDM, the majority of the target range is still being developed in this matrix. However, the primary assumption for using blood for TDM is that drug concentration in the blood compartment is reflective of that attained in the target site, which is not always the case (e.g., concentrations of antibiotics can differ in blood compared to muscle or fat tissues [1]). Moreover, the act of obtaining blood is a relatively invasive procedure which can potentially lead to untoward adverse effects such as iatrogenic infection and psychological unpleasantness [2]. In many patient populations (e.g., elderly, pediatric, anemic) and those with fragile veins, it might be impossible, impractical, or even unethical to obtain this biological fluid for any therapeutic purpose. With the substantial costs associated with collecting and analyzing blood (e.g., salary for nurses, phlebotomists, and lab technicians), it is of great interest for researchers and clinicians to find alternative biological matrices that can be as effective as blood for TDM but without the aforementioned untoward effects.

While many non-blood biological matrices have been investigated in the context of TDM [3], many have limitations that preclude their routine usage in the clinic. One of the few exceptions is interstitial fluid (ISF), which has gained a significant interest and recently fueled the research activities of many laboratories, including ours [1,4,5,6]. In the context of finding an alternative biological matrix for TDM, this manuscript will provide a qualitative review on: (1) the principles of TDM; (2) alternative matrices for TDM; (3) current evidence supporting the use of ISF for TDM in clinical models; (4) the use of microneedle technologies, which is potentially minimally invasive and pain-free, for the collection of ISF; and (5) future directions.

## 2. Principles of Therapeutic Drug Monitoring

The term therapeutic drug monitoring is often used interchangeably with clinical pharmacokinetic monitoring. TDM emerged as a clinical discipline in the late 1960s and early 1970s and involves the measurement and interpretation of drug concentrations in order to provide safe and efficacious dosing of drugs. In the past, we applied 4 basic principles to ascertain whether or not to perform routine TDM. These fundamental tenets were: (1) Is there a good relationship between drug concentration and pharmacological response? (2) Does wide interpatient variation exist in drug absorption, distribution, metabolism or excretion? (3) Does the drug have a narrow therapeutic range? and (4) Is the drug’s pharmacological response not readily assessable [7,8]?

Given more recent focus on patient-centered care and individualization of dosage regimens as well as judicious use of limited resources, we added to these 4 basic principles and developed a 9-step decision-making algorithm to ascertain whether or not to perform selective TDM for a given patient in a specific scenario. The 9 steps include: (1) Is the patient on the best drug for his/her specific disease state and specific indication? (2) Can the drug be readily measured in the desired biological matrix? (3) Has a good relationship between drug concentration and pharmacologic response been reported in pharmacokinetic studies conducted in humans? (4) Does this relationship still apply to the patient’s specific disease state and specific indication? (5) Is the drug’s pharmacologic response not readily assessable? (6) Does the drug have a narrow therapeutic range for the specific disease state and indication? (7) Are the pharmacokinetic parameters unpredictable, due to either intrinsic variability or the presence of other confounding factors? (8) Is the duration of drug therapy of a sufficient length for the patient to benefit from clinical pharmacokinetic monitoring? and (9) Will the results of the drug assay make a significant difference in the clinical decision-making process? (i.e., provide more information than sound clinical judgement alone) [7]? If one can answer “yes” to the last question (i.e., quintessential step 9) and “yes” to most of the other questions, then selective TDM is warranted for that particular patient scenario.

From the practical stand point, TDM is usually conducted at steady-state conditions using one or more concentrations. A trough concentration is often used as a surrogate to the area-under-the concentration time curve (AUC), the best marker for drug exposure. Limited sampling strategies (i.e., using 2–4 concentration samples to predict AUC) have become more widely utilized in various disease states as well (e.g., solid organ transplant [9]). Theoretically, once the basic PK characteristics about a drug are known, TDM can be tailored to already-established fundamental PK models (e.g., one, two, three-compartments; oral vs. parenteral; intermittent vs. continuous infusion; linear vs. non-linear (Michaelis-Menten) disposition; well-stirred vs. parallel-tube, etc.). Moreover, a universally accepted idea is that only the free drug is pharmacologically active and subjected to further PK processes (e.g., metabolism or elimination). Because free drug concentrations are not easily accessible, another branch of TDM is the development of static models to “predict” free from total drug concentrations in specific patient populations (e.g., phenytoin [10,11]). Recent advances in physiologically-based PK modeling and population PK modeling [12,13] with Bayesian applications (e.g., so-called “dynamic models” [9]) have further revolutionized the prediction/simulation of drug concentrations for TDM as well.

Since the publication of the decision-making algorithm in 1998, many clinician researchers have used it to critically assess currently available literature and determine the utility of TDM for specific drugs in specific patient scenarios [7]. Examples of papers in various therapeutic classes and special populations are available: central nervous system disorders (e.g., [14,15]), infectious disease (e.g., [16,17]), organ transplant (e.g., [18,19]), pediatric patients (e.g., [20,21]), cancer (e.g., [22]), psychiatric disorders (e.g., [23,24]). This paper will focus on step 2—can the drug be readily measured in the desired biological matrix?

## 3. Alternative Matrices for Therapeutic Drug Monitoring

Many alternative, less-invasive biological matrices (e.g., ISF, oral fluids, hair, sweat, tears, semen, breast milk, urine) have been investigated for the purpose of TDM [3,25]. In our opinion, with exceptions of a few specific applications where a certain matrix (e.g., tears) is preferred in unique situations (e.g., drug concentrations in eye disorders) [25], only saliva and ISF show the potential to replace blood primarily because drug concentrations can be quantified reliably and consistently in these matrices.

The science and practice of saliva TDM is still undergoing significant evolution [26,27,28,29]. Although saliva can be used for the monitoring of illicit drug use (i.e., “doping”) [30], the discussion here will focus only on TDM. Saliva is composed of aqueous fluids secreted by three salivary glands [28]. The composition of saliva, with respect to electrolytes and macromolecules, can differ based on intrinsic (e.g., healthy vs. diseased) and extrinsic (e.g., concurrent drugs that modulate the sympathetic or parasympathetic systems; method of saliva simulation) factors [27,28]. A common observation, however, is that protein content is significantly lower in saliva compared to blood [27,28]. There are many advantages and disadvantages of using saliva for diagnostics, which have been extensively reviewed [26,27,28,29]. For the purpose of TDM, the primary advantages for using saliva are the non-invasive nature for obtaining this biological fluid and the fact that saliva drug concentration reflects the free (i.e., pharmacologically active) concentration. Because of the latter characteristic, saliva has the distinct benefit over other matrices (e.g., whole tissue, blood) which are protein-rich and require additional analytical techniques to determine free drug concentrations. The primary disadvantages for using saliva for TDM are potential contaminants, drug instability, and lack of phase II metabolites [3,29]. In order for saliva to replace blood for the purpose of TDM, correlations between saliva-blood concentrations also need to be established.

The biochemical properties of xenobiotics (i.e., ionizability, molecular weight, lipophilicity, protein binding) are known to affect the distribution/excretion of drug into saliva [31]. Of these, the ionizability of the drug molecule in saliva appears to play a primary role in the penetration ratio because only unionized drugs can partition into this matrix [29]. Based on these principles, not all drugs are detectable in saliva. The first documented salivary TDM dates back to the 1970s when theophylline [32], digoxin [33], lithium [34], and various anticonvulsant drugs [35] were initially investigated. Since then, the suitability of conducting TDM in saliva for drugs in various therapeutic classes has been reported. While not the primary objective of this paper, the most recent review articles—based on therapeutic classes—on salivary TDM are cited here for the reader’s reference: antibiotics [36], anticonvulsants [37], antiretroviral agents [38], and psychotropics [39].

Similar to saliva, ISF has very similar composition as plasma but lower protein content [40]; therefore, drugs are mostly present in ISF in the free (active) form and the matrix is relatively easier to assay compared to blood. Because ISF serves as the connection between vasculature and cells, it acts as a conduit or medium for various signaling molecules and cellular wastes [41], although the exact composition of ISF can differ between organs or locations [40]. In addition to TDM, ISF has been used for other purposes such as diagnostics (e.g., for monitoring the degree of traumatic brain injury) [40] or treatment (e.g., being the primary site for many bacterial infections) [1]. The use of ISF for TDM can have several advantages: potentially pain-free (to be discussed below in the section on sampling techniques, including the use of microneedles), less cumbersome analytical assays (because the matrix is relatively clean and devoid of large macromolecules or cellular debris), and potentially less costly because the collection does not require specially trained personnel. Similar to saliva, however, in order for ISF to replace blood for the purpose of TDM, correlations between ISF-blood concentrations need to be established. On the other hand, unlike saliva, factors influencing the partitioning of drugs between blood and ISF are relatively poorly understood. Although biochemical properties (e.g., ionizability, molecular weight, lipophilicity, protein binding) can theoretically affect drug distribution into ISF, no good correlations have been observed with most of these variables in the currently available literature [1]. Although data are available on drug distribution kinetics (including model-based predictions) into whole tissues (e.g., [42,43,44,45]), the ISF compartment would exhibit different physiological properties and warrant an investigation itself. The currently available evidence supporting the use of ISF for TDM in humans is presented in the next section.

## 4. Current Evidence Supporting the Use of Interstitial Fluid for Therapeutic Drug Monitoring in Clinical Models

For the purpose of this manuscript, data pertaining to cerebral spinal fluid, brain extracellular fluid, tumor ISF, or any internal organ tissue fluid which is not routinely sampled and cannot be characterized non-invasively for the purpose of TDM will be excluded. This approach is similar to exclusion criteria we published previously [1]. Likewise, only studies incorporating microdialysis have been included, because other models (skin blisters, whole tissue) do not adequately reflect the ISF space [1]. Based on our comprehensive investigation in an animal model using an extensive panel of drugs commonly subjected to TDM today [4], a novel scoring algorithm was developed to characterize the suitability of conducting TDM in ISF: directly suitable (“comparable exposure and similar concentration-time profile versus blood”); likely suitable (“different exposure, but similar concentration-time profile versus blood”); unlikely suitable (“detectable in ISF, but different concentration-time profile versus blood”); and not suitable (“not detected in ISF”) [4]. We have already used this algorithm to analyze the suitability of using ISF or saliva for TDM in humans for anti-infective drugs [1,36]. The same principles will be employed in this paper to discuss the evidence supporting the use of ISF for TDM of other drug classes. In short, the current state of knowledge on the use of ISF for TDM in humans is still limited. A thorough literature review indicates that only a few drug classes have been investigated (i.e., anti-infectives, anticonvulsants, and miscellaneous other agents). A body of literature is also available on the measurement of drug concentrations in solid tumors [46,47,48,49,50,51,52] which is excluded from this paper (i.e., not used routinely for the purpose of TDM) and has been reviewed elsewhere [53,54].

### 4.1. Anti-Infectives

The majority of the ISF data collected in humans to date is related to anti-infectives because ISF is the primary target compartment of interests for various types of infections [1]. The evidence supporting the use of ISF for TDM for antibiotics has been systematically reviewed by Kiang et al. up to February 2014 [1], which will be summarized briefly. Additional novel data published since this paper will be presented in more detail.

Kiang et al. [1] provided a comprehensive review detailing 87 individual PK comparisons between tissue ISF and blood on various antibiotic classes: penicillins, cephalosporins, fluoroquinolones, carbapenems, oxazolidiones, macrolides, glycopeptides/glycylcycline/lipopeptide and other agents. Overall, different antibiotics exhibited different degrees of penetration into ISF of muscle or adipose tissue [1]. With respect to penicillins, the majority of data were collected on piperacillin, which showed reduced concentration in ISF compared to blood, especially in critically ill (i.e., intensive care) patients. The ability of a specific cephalosporin (i.e., cefaclor, cefixime, cefodizime, cefpirome, cefpodoxime, ceftobiprole, cefuroxime) to penetrate into ISF also appears to be dependent on the type of tissue (i.e., muscle vs. adipose) and the degree of protein binding. In the case of cefpirome, a significant reduction in ISF penetration was observed in critically ill patients, indicating that a potentially more aggressive dosing regimen might be warranted in this patient population [1]. More recently, Roberts et al. [55] compared the pharmacokinetics of cefazolin in subcutaneous ISF and blood in post-trauma critically ill patients (*N* = 30) and found similar exposure (median % penetration ratio of 74%) between the two matrices. Despite the fact that specific pharmacokinetic values were not reported, the concentration-time profiles between the two matrices appear approximately “equivalent”, indicating that it is suitable to monitor cefazolin in ISF in this population (albeit cefazolin concentrations are not typically measured in clinical practice). Overall, most of the penicillins and cephalosporins can be categorized as likely suitable drugs for TDM in ISF, due to comparable elimination characteristics but different exposure values in both blood and ISF.

Different degrees of tissue ISF penetration were observed for carbapenems in humans [1]. While doripenem and ertapenem show consistent distribution ratios between different tissues, inconsistent findings were reported for imipenem between muscle and adipose and between studies. The inconsistencies found with imipenem may have been attributed to experimental artifacts, pointing to the potential limitations of comparing findings from multiple sources utilizing different microdialysis calibration techniques. Moreover, imipenem’s distribution into ISF in critically ill patients might be dependent on renal function, as evident by contrasting findings shown by Tegeder et al. [56] and Dahyot et al. [57]. More recently, Varghese et al. [58] determined the penetration ratio of meropenem in critically ill patients receiving continuous hemodialysis and noted reduced exposure in subcutaneous ISF (60–74%) compared to blood. Although it was not clear whether the free blood concentration was determined in this study, the concentration-time profiles of meropenem in both matrices exhibited similar characteristics. Overall, the carbapenems can be categorized as likely suitable for TDM in ISF, due to comparable elimination characteristics but different exposure values in both blood and ISF [1].

With respect to the glycopeptides/glycylcycline/lipopeptides, both daptomycin and tigecycline distribute into tissue ISF completely in a manner independent of tissue inflammation or the diagnosis of diabetes [1]. These data suggest that daptomycin and tigecycline could be likely suitable for TDM in ISF (although they are not subjected to routine TDM today). Although vancomycin distributes well into tissues (penetration ratio of 0.8 from comparable exposure in two matrices based on free concentrations) in otherwise healthy subjects with limb infections [59], evidence suggests a reduced penetration in diabetic patients [60,61]. Population modeling also suggests potential suboptimal vancomycin tissue exposure in diabetics which may result in treatment failure [60], but these simulations require further testing in real clinical subjects. The findings for vancomycin in humans of comparable pharmacokinetic characteristics between ISF and blood is consistent with that observed in various animal models, including that published in our own lab [1,62]. Because vancomycin is one of the most frequently monitored drugs in the clinic today, these data suggest that vancomycin could be a potential drug candidate to test the paradigm of using ISF for the purpose of TDM. However, more studies are needed to characterize the effects of intrinsic and extrinsic factors that may affect the disposition characteristics of vancomycin. Further pharmacokinetic modeling is also needed to determine the relationship between ISF and blood concentrations, tailored for the purpose of TDM.

In contrast to the heterogeneity of tissue ISF penetration characteristics observed within many antibiotic classes presented in this manuscript, some consistent class effects of robust tissue distribution could be observed for fluoroquinolones (ciprofloxacin, levofloxacin, moxifloxacin, except for gemifloxacin) and oxazolidinones (linezolid and torezolid) [1], because the distribution ratios appear complete and independent of tissue type (muscle vs. adipose) or disease state (healthy vs. sepsis or diabetes). On the other hand, many macrolide antibiotics (except for telithromycin) penetrate into tissue ISF relatively poorly, resulting in potentially suboptimal drug concentrations at the target site [1]. Based on these characteristics and the available limited data, fluoroquinolones and oxazolidinones in general might be suitable for TDM in ISF whereas the macrolides would not be suitable. While the clinical utility of fluoroquinolone or oxazolidinone TDM still remains to be established, further research is needed to elucidate how drugs in certain classes penetrate into ISF space better than others and careful consideration is needed when selecting and avoiding antibiotic classes for the treatment of tissue infections.

### 4.2. Anticonvulsants

There is a significant interest regarding TDM for anticonvulsants using alternative body fluids [37,63] and ISF is no exception. The majority of the experiments pertaining to ISF have been conducted with valproic acid [64,65,66,67] although preliminary data on other agents (topiramate, carbamazepine, phenytoin, and phenobarbital) are also available [68,69]. Given that patients taking these agents are often monitored on an outpatient basis, having a TDM matrix that is more convenient than blood could potentially improve patient care and quality of life. Although these authors indicated collecting extracellular fluid, their approach (i.e., in vivo microdialysis on subcutaneous tissue) is reflective of free drug concentrations in ISF. The data for valproic acid look promising in that there is a good qualitative correlation between free tissue and blood concentrations in epileptic [64] and healthy subjects [65]. Moreover, the concentration-time profiles of free valproic acid in epileptic patients (*N* = 3) receiving a single dose of valproic acid or in one subject under steady-state conditions were virtually superimposable between ISF and blood [66]. However, a subsequent study using a larger sample size indicated a higher free fraction in plasma under steady-state conditions compared to single-dose conditions [67]. Although specific pharmacokinetic parameters were lacking in these studies to allow a systematic categorization of suitability, these data are suggestive of the general appropriateness of using ISF for TDM for valproic acid. Given that valproic acid is a frequently monitored anticonvulsant drug today, further systematic studies are needed to establish the quantitative relationships between ISF and blood (i.e., using compartmental, population modeling) under various conditions (e.g., single-dose vs. steady-state, healthy vs. various diseased populations requiring valproic acid, mono-therapy vs. polypharmacy, etc.) in order to determine the role of ISF for TDM.

In a single epileptic patient receiving doses of topiramate (not under steady-state conditions) co-administered with phenytoin and dextropropoxifen, the concentration-time profiles of free topiramate in subcutaneous ISF and blood were comparable with a high correlation coefficient (0.99) [69]. Likewise, in single case studies of patients undergoing tapering regimens, carbamazepine and phenobarbital [68] also exhibited similar pharmacokinetic characteristics in subcutaneous ISF and blood up to 70 h post-dose. In Lindberger et al. [68], phenytoin was not detectable in tissue ISF due to extensive binding to the microdialysis tubing. The observation of likely suitable nature with phenobarbital is consistent with our observation in rabbits [4]. Although we noticed the same effects (lack of detection in ISF) with phenytoin in our rabbit experiments, it was not due to recovery, indicating other factors that may prevent phenytoin’s distribution into ISF should also be considered. Unfortunately, neither study provided quantitative pharmacokinetic data to allow a systematic determination of suitability of conducting TDM in ISF for these anticonvulsants. Given that topiramate drug concentrations can be used occasionally to guide dosing in refractory, resistant seizure cases, and that phenobarbital and carbamazepine are routinely monitored, more studies in larger patient samples are warranted.

### 4.3. Miscellaneous Agents

The concentrations of various other drugs have been determined in ISF and compared to blood. Boschmann et al. [70] compared the concentrations of aliskiren in obese hypertensive subjects (*N* = 10) in adipose ISF, skeletal muscle ISF, and blood under steady-state conditions. Aliskiren appeared to distribute to skeletal muscle ISF (concentration comparable to blood) to a greater extent compared to adipose ISF. Because pharmacokinetic parameters were not characterized in this study, it was not possible to determine the suitability of conducting TDM in ISF for aliskiren. The concentration-time profile of a single healthy subject administered caffeine was illustrated by Stahle et al. [71]. Caffeine presented in the ISF at higher concentrations than blood (i.e., increased exposure), suggesting that additional mechanisms other than simple diffusion might be responsible for its distribution into the tissue compartment. Based on the five individual ISF concentration-time profiles presented in the study, it appeared that the pharmacokinetics of caffeine exhibit large variability in ISF; however, similar information was not provided in blood to draw any correlations. Pharmacokinetic parameters needed for the assessment for suitability of TDM in ISF were not presented for caffeine by Stahle et al. [71]. Stetina et al. [72] characterized the concentration-time profiles of scopolamine (after a single intravenous dose) in adipose tissue ISF and blood in healthy male subjects (*N* = 6) and found similar pharmacokinetic characteristics between the two matrices. Despite reduced maximum concentration and delayed time-to-reach maximum concentration, the overall exposure of scopolamine between the two matrices was essentially the same, as evident by a distribution ratio approaching unity. These characteristics suggest that scopolamine can potentially be a suitable agent to be monitored in ISF, although TDM for scopolamine is not currently clinically indicated.

## 5. Microneedle Technologies for Interstitial Fluid Collection for Therapeutic Drug Monitoring

Microneedle technologies have been used in non-clinical and clinical settings for their TDM, diagnostics, and physiological health monitoring using ISF rather than blood, as described here. ISF is trapped in its extracellular matrices of skin [73] leading to difficulties in extracting in biosensing applications. Considering limited availability of ISF in the skin (~20 nL mm^−2^ in the epidermis and ~800 nL mm^−2^ in the dermis [73]), extracting large volumes of ISF for bioanalysis is a major hurdle in developing sensors that rely on ISF extraction. Development of microneedle-integrated biosensors can potentially provide commercially feasible solutions to TDM and diagnostics [74]. A summary of microneedle strategies used for ISF collection and TDM is presented in Table 1.

### 5.1. Interstitial Fluid Extraction Devices for Off-Device Analysis

**Figure 1:**
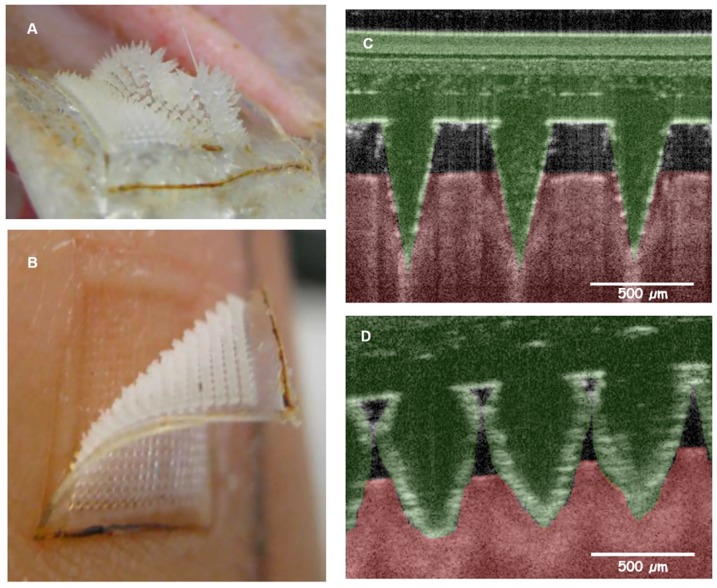
Hydrogel-forming microneedles for extraction of analytes from ISF. Figure obtained from Caffarel-Salvador et al. with permission [78]. A hydrogel forming microneedle array (**A**) before application into the skin and (**B**) after application where microneedles are swollen. Optical coherent tomography images show the microneedle arrays after skin insertion (**C**) before swelling, and (**D**) after swelling.

Several studies have successfully demonstrated techniques for ISF extraction from the skin, followed by analysis of its content outside the ISF-collecting microneedle device (hereafter termed “off-device analysis”). For example, Wang et al. [75] used a glass microneedle device to penetrate 0.7–1.5 mm into the skin in hairless rats and healthy adults, and collect 1–10 µL of ISF using a 200–500 mm Hg vacuum for 2 to 10 min for glucose measurement. Sakaguchi et al. [76] overcame vacuum-assisted extraction by applying a solid microneedle array to create micropores on the skin surface followed by a hydrogel patch to collect ISF by swelling action for glucose and sodium ion concentration measurement. Donnelly et al. [77] developed a hydrogel-forming microneedle array, using blends of hydrolyzed poly(methyl-vinylether-*co*-maleic anhydride) and polyethylene glycol crosslinked by esterification, which increased its mass when applied to skin, due to ISF uptake by the hydrogel. Caffarrel-Salvador et al. [78], Romanyuk et al. [79], and Chang et al. [83] showed that extremely small volumes of ISF (<20 µL) can be extracted by similar hydrogel-forming swellable microneedle-arrays over 1 to 2 h in porcine, human (Figure 1) [78], rat [79], and mice [83] skins.

Research conducted to date have failed to demonstrate commercial feasibility of ISF extraction followed by off-device analysis for various reasons. These include prolonged ISF extraction time, need for multiple steps between sample extraction and bioanalysis, need to further extract and/or transfer the fluid from the collection apparatus to a separate site for analysis, extremely low volume of ISF collected, and evaporation of ISF leading to measurement variability and error [74].

### 5.2. Interstitial Fluid Extraction for On-Device Analysis

Microneedle-integrated biosensors were built to extract ISF and perform the biomolecule analysis on-device, with a key feature of not needing to transfer ISF to a separate instrument. Mukerjee et al. [80] devised a hollow silicone microneedle-integrated system to collect water, glycerol, ISF, and whole blood. ISF was extracted in vivo from human earlobe skin over a 15 to 20-min period, and analyzed qualitatively for glucose [80]. Zimmerman et al. [84] developed a hollow silicon microneedle array integrated into an enzymatic glucose sensor with a porous dialysis membrane, and showed a significant sensor response after exposure to ISF ex vivo. Strambini et al. [81] developed a similar glucose sensor with hollow silicon-dioxide microneedle arrays (1 × 10^6^ needles cm^−2^) connected to chip consisting screen-printed enzymatic glucose sensor integrated to the backside of the microneedle array for ISF collection and bioanalysis. Ex vivo evaluation showed rapid detection of glucose [81]. Although more commercially feasible than ISF collection devices for off-device analysis, devices with integrated on-device analytical capabilities consist of some challenges, including the need for microliter-level volumes of ISF for analysis, prolonged time to collect ISF, and transfer of ISF to the backside of the microneedle for bioanalysis.

**Figure 2:**
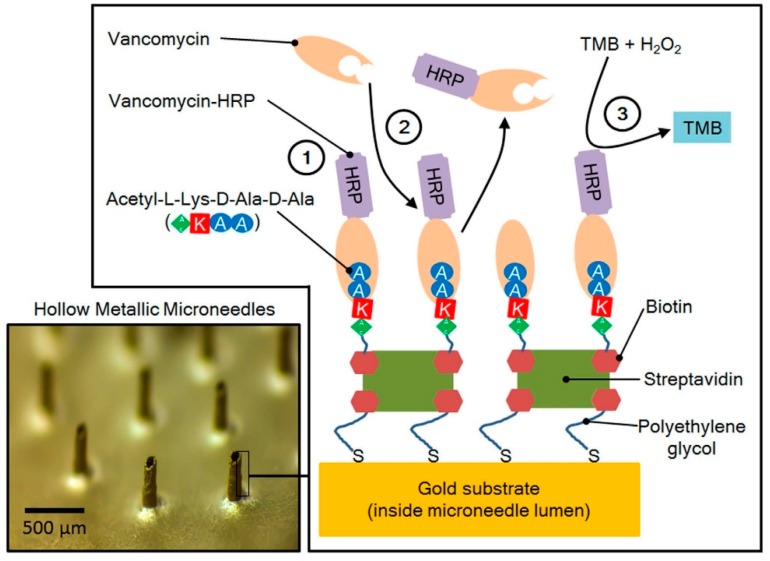
Functionalization of gold-coated hollow microneedle lumens for fluid collection, drug binding, and detection. Figure obtained from Ranamukhaarachchi et al. with permission [6].

Ranamukhaarachchi et al. [6] developed a point-of-care TDM device by integrating a gold-coated hollow microneedle with an optofluidic sensing system to detect vancomycin in sub-nanoliter volumes of fluid in vitro. Vancomycin receptors were immobilized inside the microneedle lumen (Figure 2), which collected 0.6 nL of sample (i.e., ISF) within seconds, and caused vancomycin to bind to the microneedle lumen for quantification.

The extremely small sample volume, capability to detect low concentration TDM drugs, rapid operation from collection to analysis (altogether less than 5 min TDM time), and lack of need for sample transfer from the collection site to analytical site were key advantages of this system. However, this system needs to be validated in pre-clinical and clinical studies [74].

### 5.3. Continuous Monitoring Microneedle Devices

**Figure 3:**
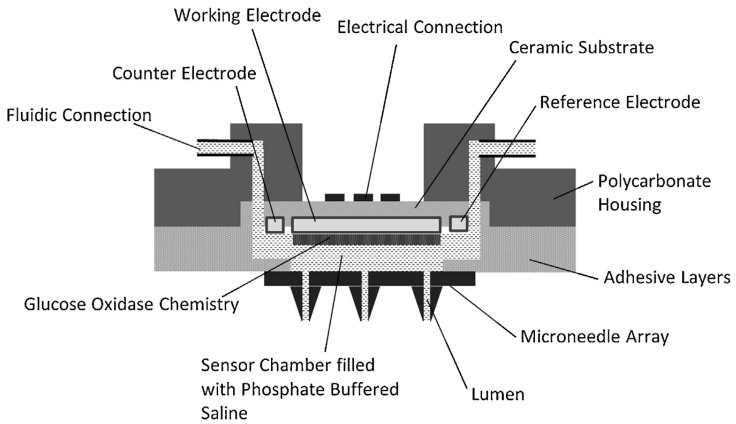
Continuous glucose monitoring biosensor. Figure obtained from Chua et al. with permission [85].

The most promising applications for microneedle-integrated biosensors is continuous monitoring of biomolecules from the skin’s ISF. Jina et al. [82] developed a hollow microneedle-integrated continuous glucose monitoring (CGM) biosensor, and demonstrated accurate and continuous glucose measurements in humans using ISF up to 72 h. The sensing chamber (Figure 3), located behind the microneedle array, was filled with a buffer solution to transfer glucose passively from ISF to sensor, eliminating the need for removal of ISF from the skin [82]. The main advantages of this system are lack of volume requirement for ISF extraction, real-time bioanalysis, and limited number of steps to obtain bioanalytical results [74].

### 5.4. Other Methods for ISF Extraction

Microdialysis catheters can be implanted in or under the skin and used to extract biomolecules, such as glucose, from ISF [86]. Other systems such as ultrafiltration [87], reverse iontophoresis [88], and sonophoresis [89] have all been used previously to extract ISF as well, but their potential is limited compared to more minimally-invasive technologies such as microneedle-based systems.

## 6. Future Directions

The conventional paradigm of monitoring drug concentrations in blood might be impractical, costly, and sometimes unethical in certain patient populations (e.g., pediatric, elderly, or those with “bad” veins). A new way of conducting TDM using ISF is gaining momentum in the research community and, in our opinion, is at the stage where its clinical utility should be investigated. In this review paper, we have presented (1) the principles of TDM; (2) alternative matrices for TDM; (3) current evidence supporting the use of ISF for TDM in clinical models; and (4) the use of microneedle technologies, which is potentially minimally invasive and pain-free, for the collection of ISF. Based on these advancements, the immediate future direction for this area of research is to establish the suitability of using ISF for TDM in human models for drugs that have been found “suitable” in pre-clinical experiments. In order to do this, a systematic approach involving compartmental modeling, physiologically-based modeling, and population pharmacokinetic modeling would have to be utilized. The effects of intrinsic and extrinsic factors that can potentially affect the pharmacokinetics of particular drugs in ISF should be incorporated into these clinical studies to fully capture the covariates and variabilities associated with this approach. In the more distant future, microneedle devices, such as those described in this review, that are minimally invasive and potentially pain-free should be tested on humans to determine their utility in the collection of ISF. Ultimately, we envision point-of-care devices where pain-free ISF collection (via a microneedle-like device), drug concentration assessment, and dosing can be done in real time, at the bedside.

## Figures and Tables

**Table 1:** Summary of microneedle strategies used for interstitial fluid (ISF) collection and therapeutic drug monitoring.

Microneedle Device Strategy	Description	Indication	Testing Model	ISF Volume Collected/Required
ISF extraction and off-device analysis	Glass hollow microneedle (0.7–1.5 mm long) and vacuum-assisted ISF collection [75]	Glucose	Tail vein of rats, finger tips of humans	1–10 µL
	Dissolving microneedle “poke” followed by vacuum suction [62]	Vancomycin	Male Wistar rats	2 µL
	Solid microneedle arrays “poked” the skin, and hydrogel “patch” collected ISF [76]	Glucose and sodium ion concentration	Human subjects	<10 µL
	Hydrogel forming microneedle array [77,78,79]	Theophylline, caffeine, glucose	Pigs, rats, and human subjects	-
ISF extraction and on-device analysis	Hollow microneedles with integrated ISF collection reservoir [80]	Glucose	Human subject	-
	Hollow microneedle array integrated with screen-printed enzyme sensor [81]	Glucose	In vitro bench testing	10 µL
	Hollow microneedle integrated with optofluidic sensor [6]	Vancomycin	In vitro bench testing	0.6 nL
Continuous ISF monitoring	Hollow microneedle integrated with buffer-filled glucose sensor [82]	Glucose	Human subjects	-
